# Analysis of *Haloferax mediterranei* Lrp Transcriptional Regulator

**DOI:** 10.3390/genes12060802

**Published:** 2021-05-25

**Authors:** Laura Matarredona, Mónica Camacho, María-José García-Bonete, Belén Esquerra, Basilio Zafrilla, Julia Esclapez, María-José Bonete

**Affiliations:** 1 Agrochemistry and Biochemistry Department, Biochemistry and Molecular Biology Area, Faculty of Science, University of Alicante, Ap 99, 03080 Alicante, Spain; lauramata1996@gmail.com (L.M.); camacho@ua.es (M.C.); belen.esquerra@ua.es (B.E.); basilio.zafrilla@ua.es (B.Z.); mjbonete@ua.es (M.-J.B.); 2 Department of Medical Biochemistry, Institute of Biomedicine, University of Gothenburg, 40530 Gothenburg, Sweden; mj.garcia.bonete@gmail.com; 3 Physiology, Genetics and Microbiology Department, Microbiology Area, Faculty of Science, University of Alicante, Ap 99, 03080 Alicante, Spain

**Keywords:** homologous overexpression, his-tag, *Archaea*, *Haloferax mediterranei*, Lrp, β-galactosidase assay, western blot, stress

## Abstract

*Haloferax mediterranei* is an extremely halophilic archaeon, able to live in hypersaline environments with versatile nutritional requirements, whose study represents an excellent basis in the field of biotechnology. The transcriptional machinery in *Archaea* combines the eukaryotic basal apparatus and the bacterial regulation mechanisms. However, little is known about molecular mechanisms of gene expression regulation compared with *Bacteria*, particularly in Haloarchaea. The genome of *Hfx. mediterranei* contains a gene, *lrp* (HFX_RS01210), which encodes a transcriptional factor belonging to Lrp/AsnC family. It is located downstream of the glutamine synthetase gene (HFX_RS01205), an enzyme involved in ammonium assimilation and amino acid metabolism. To study this transcriptional factor more deeply, the *lrp* gene has been homologously overexpressed and purified under native conditions by two chromatographic steps, namely nickel affinity and gel filtration chromatography, showing that Lrp behaves asa tetrameric protein of approximately 67 kDa. Its promoter region has been characterized under different growth conditions using *bgaH* as a reporter gene. The amount of Lrp protein was also analyzed by Western blotting in different nitrogen sources and under various stress conditions. To sum up, regarding its involvement in the nitrogen cycle, it has been shown that its expression profile does not change in response to the nitrogen sources tested. Differences in its expression pattern have been observed under different stress conditions, such as in the presence of hydrogen peroxide or heavy metals. According to these results, the Lrp seems to be involved in a general response against stress factors, acting as a first-line transcriptional regulator.

## 1. Introduction

Haloarchaea are microorganisms belonging to the *Archaea* domain characterized by high salt requirements, around 10–35% (*w*/*v*) for optimal growth [1,2]. In recent decades, these microorganisms attracted scientific attention due to the potential applications of their proteins, enzymes, and different secondary metabolites for biotechnological and industrial purposes [3]. Previous research has pointed out that *Hfx. mediterranei* is one of the most known Haloarchaea and is considered a model organism to study nitrogen metabolism due to its knowledge in terms of molecular biology and biochemistry [4]. Although enzymes from nitrogen metabolism pathways have been previously studied in this haloarchaeon, little is known about the genetic regulation of these pathways compared with Bacteria. Therefore, more research is needed to elucidate the molecular mechanism of transcriptional regulator in *Hfx. mediterranei.*

There is little information about the molecular mechanisms of gene expression regulation in members of the *Archaea* domain. The genetic manipulation is still limited compared to the *Bacteria* domain, particularly in halophilic microorganisms. Few researchers have been focused on figuring out the function of transcriptional regulators. The transcriptional machinery in *Archaea* combines the eukaryotic basal apparatus and the bacterial regulation mechanisms. One group of archaeal transcriptional regulators is the leucine-responsive regulatory protein/asparagine synthase C family (Lrp/AsnC), also known as feast/famine regulatory proteins (FFRPs) [5]. Members of this family influence the metabolism globally (Lrp) or specifically (AsnC). The Lrp/AsnC family is the most abundant in archaeal genomes, being represented in almost sequenced genomes to date [6,7]. Apart from *Archaea*, these proteins have also been identified in members from *Bacteria* [8]. Members of the family Lrp/AsnC are small DNA-binding proteins containing two domains: the DNA-binding domain and the ligand-binding domain. The DNA-binding domain is also known as helix-turn-helix (HTH) domain. It is located in the N-terminal part of the protein, and it is responsible for the specific DNA interaction. The C-terminal region contains a ligand-binding domain known as the regulation of amino acid metabolism domain (RAM), facilitating the effector binding and/or its oligomerization [8,9]. The most extensively studied protein from this family is an Lrp from *E. coli* which acts as a global regulatory protein controlling a regulon encompassing more than 400 genes [10]. This family is considered one of the best-studied families. Indeed, there are previous investigations about proteins from archaeal model organisms such as *Sulfolobus*, *Pyrococcus*, *Methanocaldococcus*, and *Halobacterium* [11,12,13,14,15].

Although the information of Lrp/AsnC in Haloarchaea is limited in comparison with bacteria, there is a previous work focused on Lrp-like regulators, LrpA1 and Lrp, in *Hbt. salinarum* R1. This work demonstrates that Lrp activates the gene expression of the *glnA* gene, influences the peptide and phosphate transport, and participates in the central intermediary metabolism acting as a global transcriptional factor [12]. Lrp acts as a global regulator affecting amino acid metabolism regulation, while LrpA1 has a specific regulatory function targeting an aspartate transaminase gene [12]. Indeed, *Hfx. mediterranei* has several genes that encode Lrp/AsnC transcriptional factors, and one of them, *lrp* (HFX_RS01210), is homologous to that of *Hbt. salinarum*. This gene is also located next to the glutamine synthetase gene, *glnA* (HFX_RS01205), an enzyme involved in ammonium assimilation and amino acid metabolism [16,17]. Therefore, this study focuses on expanding the Lrp/AsnC family’s knowledge in *Archaea* and analyzing the *lrp* gene involvement in the nitrogen cycle and under stress conditions. The Lrp has been homologously overexpressed to reach these aims, and its quaternary structure has been determined. Its expression in the presence of different nitrogen sources and stress conditions has been studied using two different approaches: promoter region characterization using *bgaH* as a reporter gene and protein amount using Western blotting.

## 2. Materials and Methods

### 2.1. Bioinformatic Analysis

Bioinformatic analysis was performed to study in-depth the Lrp/AsnC family of transcriptional regulators in Haloarchaea. The number of these proteins annotated as Lrp/AsnC, and their domain structures were analyzed using the UniProt database (https://www.uniprot.org/, accessed on 20 March 2021). Furthermore, 14 Lrp sequences from *Hfx. mediterranei* obtained from the protein database of NCBI (National Center for Biotechnology Information) (https://www.ncbi.nlm.nih.gov/protein/, accessed on 20 March 2021) were used to construct the phylogenetic tree. Alignments were performed using the software Clustal Omega (ClustalO) as a multiple sequence alignment program (https://www.ebi.ac.uk/Tools/msa/clustalo/, accessed on 20 March 2021) based on the HH algorithm described by Söding [18,19]. Then, a phylogenetic tree was built using the neighbour-joining method from Clustal Omega. The display, manipulation and annotation of the phylogenetic tree were done using the online tool known as Interactive Tree Of Life (iTol) v4 (https://itol.embl.de/, accessed on 20 March 2021) [20]. Furthermore, another sequence alignment was performed using the previous software, the Clustal Omega, with HTH-domain sequences from *Hfx. mediterranei* Lrps. Then, the Mview tool (https://www.ebi.ac.uk/Tools/msa/mview/, accessed on 20 March 2021) was used to find consensus sequences.

### 2.2. Strains, Plasmids and Culture Conditions

*Escherichia coli* strains DH5α for cloning and JM110 for preparing unmethylated DNA for efficient transformation of *Hfx. mediterranei* were grown overnight in Luria-Bertani medium with ampicillin (100 µg/mL) at 37 °C.

*Hfx. mediterranei* R4 (ATCC 33500^T^) and *Hfx. mediterranei* HM26 (R4-Δ*pyrE2*) [21] were grown at 42 °C in complex medium (Hm-CM) containing 20% (*w*/*v*) seawater (20% SW) [22] and 0.5% (*w*/*v*) yeast extract (pH 7.3).

The plasmid used for protein overexpression was pTA1992, kindly provided by Dr Thorsten Allers (University of Nottingham, UK). This vector contains pHV2 origin, *pyrE2* and *hdrB* markers to allow the selection on media lacking uracil and thymidine, and strong p.syn synthetic promoter for constitutive overexpression of halophilic proteins with a N-terminal His-tag and/or a C-terminal StrepII-tag [23,24]. The plasmid used for characterizing the promoter region was pVA315 (12359 bp), kindly provided by Dr Mike Dyall-Smith (University of Melbourne, Australia). This vector contains *E. coli* pBR322 plasmid *ori* region, ampicillin resistance (Amp^R^) gene, the *Haloferax* pHK2 replicon region and novobiocin-resistance (Novo^R^) gene, enabling maintenance and selection in both hosts. It also contains the β-galactosidase (*bgaH*) gene from *Haloferax lucentense* as a reporter gene [25,26].

*Hfx. mediterranei* minimal medium (Hm-MM) contained a concentration of 20% (*w*/*v*) seawater, 10 mM NH_4_Cl and 0.25% (*w*/*v*) casamino acids (pH 7.3). After autoclaving and cooling, it was supplemented with 50 mM MOPS (3-(N-morpholino) propane sulfonic acid) pH 7.3, 0.03 mM FeCl_3_, 1 mM KH_2_PO_4_ and 7.5 mM CaCl_2_ per litre. For solid media, agar (Conda, Torrejón de Ardoz, Madrid, Spain) was added to a final concentration of 18 g per litter. *Hfx. mediterranei* defined medium (Hm-DM) was prepared as Hm-MM, but casamino acids were replaced by 20 mM NH_4_Cl or KNO_3_ as the nitrogen source. It was supplemented as Hm-MM, and 28 mM of glucose was added as the carbon source after autoclaving. To study the effect of nitrogen starvation, *Hfx. mediterranei* nitrogen starvation medium (Hm-NS) was performed by growing Hm-DM cultures with NH_4_Cl as the nitrogen source until the mid-exponential growth phase. To induce the nitrogen starvation, cells were harvested by centrifugation during 20 min at 13,000 rpm, washed with 20% seawater, and then transferred to a medium without a nitrogen source. *Hfx. mediterranei* carbon starvation medium (Hm-CS) was performed following the same steps as in nitrogen starvation medium but transferring the cells to a medium without carbon source. Cells were subjected to nitrogen or carbon starvation for 96 h. All the culture media were incubated aerobically at 42 °C with shaking (220 rpm).

### 2.3. Homologous Overexpression of pTA1992.lrp in Hfx. mediterranei HM26

The *lrp* gene was amplified from *Hfx. mediterranei* R4 genomic DNA using the forward primer 5′-CACCACCACCACATGACGTACGAAAACCTCGATGCG-3′ and the reverse primer 5′-CGGGCTGCAGGAATTCATTCGTCGACGTCGAGCGC-3′, including restriction sites for *EcoR*I and *BamH*I (Thermo Fisher Scientific, Waltham, MA, USA), respectively. The plasmid construction and the insert were generated under the manufacturer’s instructions described in the In-Fusion HD cloning kit (Clontech, Torrejón de Ardoz, Madrid, Spain), retaining the vector’s N-terminal His_6_ tag. The resulting ligation was introduced into *E. coli* DH5α and then into *E. coli* JM110 using a standard transformation protocol [27]. Following this, *Hfx. mediterranei* HM26 cells were transformed with the construction pTA1992.*lrp* using a revised version of the protocol mediated by using polyethylene glycol 600 [28] and plated on Hm-MM agar plates. Plates were incubated at 42 °C for 5–7 days until pink colonies were visible. The transformant selection was based on the *pyrE2* marker. Selected colonies with pTA1992.*lrp* were cultured in Hm-MM and grown until the stationary phase. The culture was harvested by centrifugation at 13,000 rpm for 30 min and resuspended in ice-cold binding buffer (20 mM Tris-HCl, 1.5 M NaCl, 50 mM imidazole, pH 7.4). Cells were lysed by sonication until the suspension was no longer turbid. The cell lysate was centrifuged, and the supernatant was collected to purify the protein.

### 2.4. Protein Purification and Determination of Molecular Mass

A two steps purification was performed on an ÄKTA chromatography system (GE Healthcare Life Sciences, Cornella de Llobregat, Spain). First, the overexpressed protein was purified by nickel affinity chromatography using a prepacked *HisTrap* HP 5 mL column following the manufacturer’s indications. Bound protein was eluted in elution buffer containing 500 mM imidazole in the binding buffer. The elution fraction containing the Lrp protein was concentrated using a Vivaspin-20 with a cut off 5 kDa and loaded in Superose6 Increase 10/300 GL column previously equilibrated with 50 mM Tris-HCl buffer (pH 8.0) containing 150 mM NaCl. Standard proteins for gel filtration chromatography ranging from 6.5 to 660 kDa were used as markers to estimate the protein molecular mass (Gel Filtration Calibration Kit LMW and HMW, Cytiva Europe GMBH) using the same buffer than for Lrp protein.

All fractions were analyzed on 14% SDS-PAGE using PageRuler Plus Prestained Protein Ladder (Thermo Fisher Scientific, Waltham, MA, USA) as molecular weight markers. Proteins were detected using Coomassie Brilliant Blue staining.

### 2.5. Characterization of lrp Promoter Region Using bgaH as a Reporter Gene

The *lrp* promoter region (p.*lrp*) was amplified from *Hfx. mediterranei* R4 genomic DNA using the forward primer 5′-TTGTCTTCCGTCATTTTCCTGAACAT-3′ and the reverse primer 5′-CGCATCCATGGTTTCGTACGTCAT-3′ including restriction sites for *Hind*III and *Nco*I (Thermo Fisher Scientific, Waltham, MA, USA), respectively. This promoter region was cloned in pGEM-T Easy Vector Systems (Promega, Barcelona, Spain) and subsequently into the pVA513 expression vector. *Hfx. mediterranei* R4 cells were transformed with pVA513.*p.lrp* as was described previously, and the transformants were selected on agar plates containing 0.3 µg/mL of novobiocin. The promoter region of *lrp* was characterized in different culture media (Table 1) by measuring β-galactosidase activity at the mid-exponential phase (OD_600_ 1.5). The β-galactosidase activity was determined by using *o*-nitrophenyl-β-_D_-galactospyranoside (ONPG) and the bgaH buffer (50 mM Tris-HCl pH 7.2, 2.5 M NaCl, 10 µM MnCl_2_, 0.1% β-mercaptoethanol) [25]. Cells pellets were resuspended to 20% (*w*/*v*) in bgaH buffer and incubated for 3 min at 40 °C with ONPG. The increase in absorbance at 405 nm was recorded for 5 min to measure β-galactosidase activity. The activity measurements were performed in triplicates, and the protein concentration of extracts was determined by the Bradford assay [29]. The results of β-galactosidase activity at the mid-exponential phase were represented in graphs using GraphPad Prism (Version 8). All values in figures are expressed as the mean of three replicates ± the standard deviation.

### 2.6. Western Blot Analysis

Western blot assays were performed to analyze the abundance of Lrp in cell extracts of *Hfx. mediterranei* R4 strain in the same growing conditions described in Table 1. Pellets were resuspended to 30% (*w*/*v*) in 138 mM NaCl, 54 mM Na_2_HPO_4_·2H_2_O, 1.5 mM NaH_2_PO_4_, 3 mM KCl (pH 7.5). Protein concentrations were determined by Bradford assay. 20 µg of protein was separated in 14% SDS-PAGE, transferred onto PVDF membrane (GE Healthcare Life Sciences, Cornella de Llobregat, Spain) and probed with a primary polyclonal rabbit antibody anti-Lrp (0.3 µg/mL) (GenScript, NJ, USA). The protein of interest was detected with an anti-rabbit HRP-conjugated antibody (1:50,000) (Thermo Scientific, Waltham, MA, USA) and visualized with Amersham ECL Prime Western blotting Detection Reagent (GE Healthcare Life Sciences, Cornella de Llobregat, Spain). The overexpressed protein was used as the positive control.

## 3. Results and Discussion

### 3.1. Lrp/AsnC Transcriptional Factor in Haloarchaea

Genome analysis was performed to obtain information about the distribution and domain structure of *lrp/asnC* genes in halophilic archaea. The analysis of all halophilic archaeal families’ genomes is summarized in Table 2. In general, halophilic microorganisms encode many Lrp/AsnC proteins in their genomes; consequently, it is reasonable to think that these transcriptional factors play crucial roles in cells. The analysis of amino acid sequences revealed that most of the halophilic archaeal Lrp/AsnC proteins contain the DNA-binding domain (HTH) as well as the ligand-binding domain (RAM). That is also the typical domain structure found in the Lrp/AsnC proteins in *E. coli*. The DNA-binding domain represents the most conserved part of the amino acid sequence. These results reveal that haloarchaeal transcriptional factors comprise a significant proportion of double domain Lrp/AsnC proteins, although some proteins only contain a single domain, the HTH or RAM domain.

Furthermore, some species of these families present another catalytic motif: the TrkA_C or the TRASH domain. Both domains were not previously identified in prokaryotic proteins, and they are present in all the Haloarchaea families except in the *Halococcaceae* family. This family has the highest percentage of Lrps with the HTH and the ligand-binding domain comparing to other halophilic archaeal families. The *Halobacteriaceae* family has the highest number of Lrps containing the TRASH and TrkA_C domain with 3.5% and 3.1%, respectively. Besides, comparing the percentages of the domain structure among families, it has been found that it is more frequent to find Lrps containing the ligand-binding domain than the DNA-binding domain.

Intriguingly, it seems that the number of *lrp* genes and nutritional requirements are directly related in *Archaea*. Organisms such as methanogens, mostly autotrophically living in habitats with specific nutritional requirements, exhibit a limited number of regulators belonging to the Lrp/AsnC family. Nevertheless, archaea with a high metabolic diversity usually contain many of these transcriptional regulators [9]. *Hfx. mediterranei* is metabolically very versatile, growing using a wide range of carbon and nitrogen sources and, even, in the presence of heavy metals [30,31]. The high number of Lrp/AsnC proteins, compared with other haloarchaeal species, may allow *Hfx. mediterranei* using a wide range of nutrients.

It is necessary to determine the function of Lrp protein in regulating gene expression and discover their level of involvement in physiological and biochemical cell processes to unlock new biotechnological and industrial applications of these microorganisms.

### 3.2. Phylogeny and Lrp/AsnC Proteins Domains in Hfx. mediterranei R4

**Figure 1:**
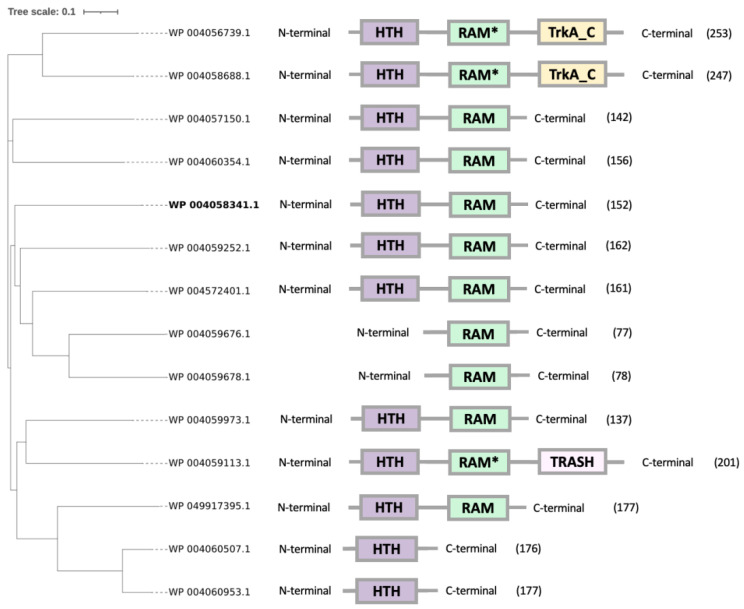
Phylogenetic tree with 14 sequences of Lrps from *Hfx. mediterranei* and their Protein domain structure. In bold the Lrp protein of interest in this study; in parenthesis the number of amino acids residues; in purple the DNA-binding domain (HTH); in green the ligand-binding domain (RAM); in yellow the TrkA_C domain; and in pink the TRASH domain. Asterisks indicate lower similarity with the query RAM domain PF01037 from PFAM.

*Hfx. mediterranei* has 14 homologs of Lrp/AsnC proteins whose phylogeny, length and domain identification of each one of them are shown in Figure 1. The average number of amino acid residues in this family is around 160. However, *Hfx. mediterranei* contains some Lrp/AsnC proteins that differ in the length expected, having longer chains (up to 247–253 amino acid residues) or shorter chains (up to 77–78 amino acid residues).

To explore the phylogenetic relationships among members of *Hfx. mediterranei* Lrp/AsnC family, specifically the Lrp protein of interest in this study (WP_004058341.1), a phylogenetic tree was constructed (Figure 1), and a total of 14 available sequences coding for Lrp/AsnC proteins were analyzed. This phylogenetic tree shows a clear and early divergence of the branches. This matches what is expected because of the low sequence conservation of this family of transcriptional regulators. The Lrp (WP_004058341.1) contains the DNA-binding domain and the ligand-binding domain.

Furthermore, it has been reported that from Lrps identified in archaeal sequences, many of them have around 160 amino acids, which are known as full length. A full-length Lrp protein is composed of an N-terminal DNA-binding (HTH) domain and a C-terminal ligand (RAM) domain. However, other archaeal Lrps have only around 80 amino acid residues known as demi Lrps, which lack the HTH domain and cannot bind to DNA [32]. According to Figure 1, most of the Lrp proteins in *Hfx. mediterranei* have the DNA-binding (HTH) domain as well as the ligand-binding (RAM) domain, but there are some exceptions: (i) two proteins containing fewer amino acid residues presenting only the ligand-binding domain (WP_004059678.1 and WP_004059676.1); (ii) two Lrp contain the DNA-binding domain (WP_004060507.1 and WP_004060953.1); (iii) three proteins containing a higher number of amino acid residues present an additional domain in the C-terminal part of the protein. This additional domain can be a TrkA_C domain (WP_004056739.1 and WP_004058688.1) or a TRASH domain (WP_004059113.1). The exact function of the TrkA_C domain remains unknown. The exact function of this domain remains unknown. However, it is predicted to bind unidentified ligands and to regulate sulfate, sodium, and other transporters [33]. The presence of the TRASH domain in the Lrp (WP_004059113.1), apart from the other two domains, suggests that it may be involved in metal coordination [34].

The results from Figure 1 can be compared with previous research about the evolution of homologous transcriptional factors belonging to the Lrp/AsnC family in *Hbt. salinarum* NRC-1, where the eight Lrps homologs are full-length, and there is another additional Lrp that contains only the ligand-binding domain, missing the DNA-binding domain [35]. Both haloarchaea have a high number of these transcriptional factors in common compared with the average sequenced archaeal genomes with 5 ± 4 homologs suggesting that progenitors from many of the Lrps were present in a common ancestor. Due to that fact, it can be explained the functional and functional divergence between the homologs Lrps from *Hfx. mediterranei.* An example of this divergence is the appearance of new domains as the TrkA_C and TRASH domain.

To deeply understand how the fourteen members of this family have diverged, it would be helpful to know how these transcriptional regulators act and which function they play. All the Lrps from *Hfx. mediterranei* will have different roles in the microorganism as they have variations in the DNA-binding domain, in the ligand-binding domain, or the effector molecule.

**Figure 2:**
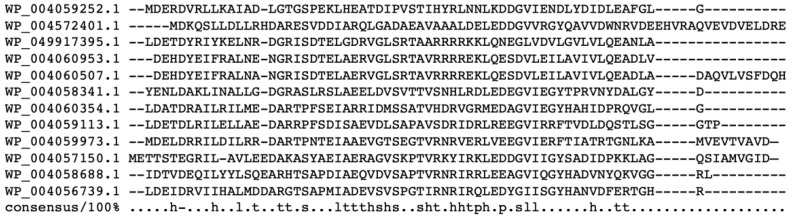
Clustal alignment with Mview of the HTH domain sequences from *Hfx. mediterranei*. Screenshot of multiple sequences alignment generated by ClustalO and viewed using Mview option. h: hydrophobic; l: aliphatic; t: turnlike; s: small; p: polar.

The most conserved part of the Lrp/AsnC proteins is the HTH domain, although their sequence conservation is only 20–30%. The Lrps from *Hfx. mediterranei* containing this DNA-binding domain were used to perform a sequences alignment using ClutalO and Mview (Figure 2) to study the consensus sequences of the HTH domain. WP_004059678.1 and WP_004059676.1 were not included because they lack the HTH domain.

### 3.3. Gene-Environment

**Figure 3:**
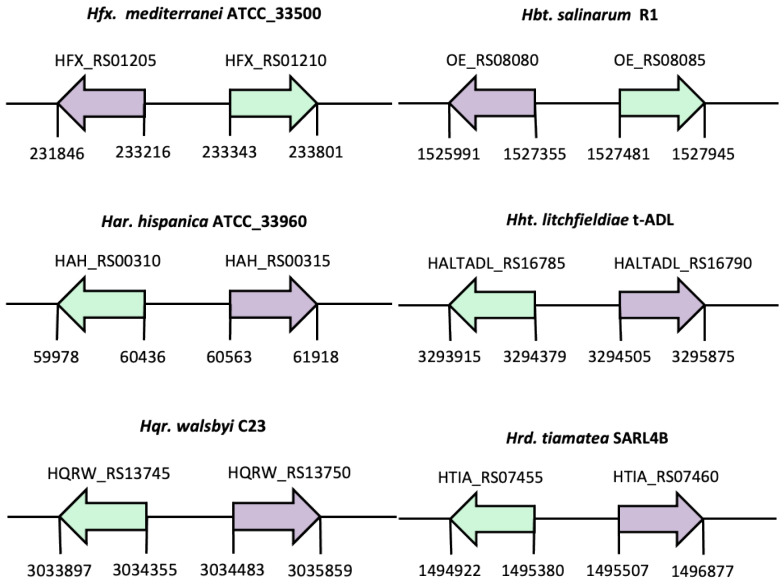
Gene-environment of *lrp* genes in halophilic organisms. In *Hfx. mediterranei* and *Hbt. salinarum*, *lrp* is located downstream of the *glnA* gene. In *Har. hispanica*, *Hht. litchfieldiae*, *Hqr. walsbyi* or *Hrd. tiamatea,* the *lrp* is located upstream of the *glnA*. Both genes are orientated in opposite directions. In green *lrp* gene and purple *glnA*.

As it has been described above, *Hfx. mediterranei* genome contains an *lrp* gene (object of this study) located next to the *glnA* gene. This gene arrangement is also conserved in *Hbt. salinarum.* Therefore, to find out if this arrangement has some influence on *glnA* gene expression in *Hfx. mediterranei*, both the *lrp* gene-environment and amino acid sequences were analyzed (Figure 3 and Supplementary Figure S1). In both species, the *lrp* gene is located downstream of the *glnA* gene in the opposite direction, having separated promoters. In other organisms, such as *Haloarcula hipanica*, *Halohasta litchfieldiae*, *Haloquadratum walsbyi* or *Halorhabdus tiamatea*, the *lrp* gene is located upstream and in the opposite direction of *glnA* (Figure 3). In these species, the Lrp also contains the HTH domain and the ligand-binding as the Lrp from *Hfx. mediterranei*. Therefore, it is typical to find the *glnA* near the Lrp transcriptional regulator in many halophiles species. Supplementary Figure S1 shows the alignment between the Lrp (HFX_RS01210) from *Hfx. mediterranei* with Lrp (OE_RS08085) from *Hbt. salinarum*. This sequence alignment has shown that the DNA-binding (HTH) domain is a conserved region, and both transcriptional regulators are very similar, with 73.4% identity and 85.1% similarity (Supplementary Figure S1). The Lrp/AsnC family is characterized by relatively low sequence conservation with a sequence identity between 20–30% [9]. However, these results considering the HTH domain show high sequence conservation. The highest identity is found in the N-terminal region, where the HTH-DNA-binding domain is located. Indeed, the degree of identity increases by analyzing only the N-terminal region of the proteins. Comparative sequence analysis of the known proteins belonging to the Lrp/AsnC family was performed using protein BLAST. The highest identity score (98.03%) among all Lrp/AsnC proteins from all organisms, and the Lrp as transcriptional regulator object of this study by comparing all the amino acid sequences, can be found in *Haloferax mucosum* (WP_008319874.1).

### 3.4. Overexpression, Purification and Determination of the Molecular Mass of Hfx. mediterranei Lrp

*E. coli* has been possibly the most used bacteria for heterologous gene expression in prokaryotes and eukaryotes [32,33,34]. However, using *E. coli* as the host for the expression of proteins from halophiles has several limitations due to the nature of these proteins, which have a high content of acidic amino acid residues aspartate and glutamate on the surface of the proteins, and high salt concentration requirements [35,36,37]. These low ionic strength limitations can cause difficulties since halophilic proteins fail to fold into their native state and aggregate into an insoluble fraction known as inclusion bodies. Therefore, few proteins from *Hfx. mediterranei* have been successfully overexpressed in *E. coli* [38,39,40,41]. The recombinant proteins are usually obtained as inclusion bodies, which are solubilized in the presence of buffers containing urea and refolded in hypersaline solutions to recover their native structure [42,43]. However, homologous overexpression using a halophilic host avoid these disadvantages. Previous works have shown a system for the homologous overexpression and purification of halophilic proteins under native conditions in the haloarchaeon *Hfx. volcanii* [44,45,46]. There is a previous attempt of homologous overexpression of Cu-NirK from *Hfx. mediterranei* using *Hfx. volcanii* as halophilic host and an expression vector with a constitutive and strong promoter [47].

Nevertheless, this is the first report of a homologous overexpression and purification of a *Hfx. mediterranei* protein in the same microorganism, using a native expression system with His-tagged, which improves purification yield and enrichment [48]. Methods for the production and purification of haloarchaeal proteins are essential for subsequent biotechnological applications.

**Figure 4:**
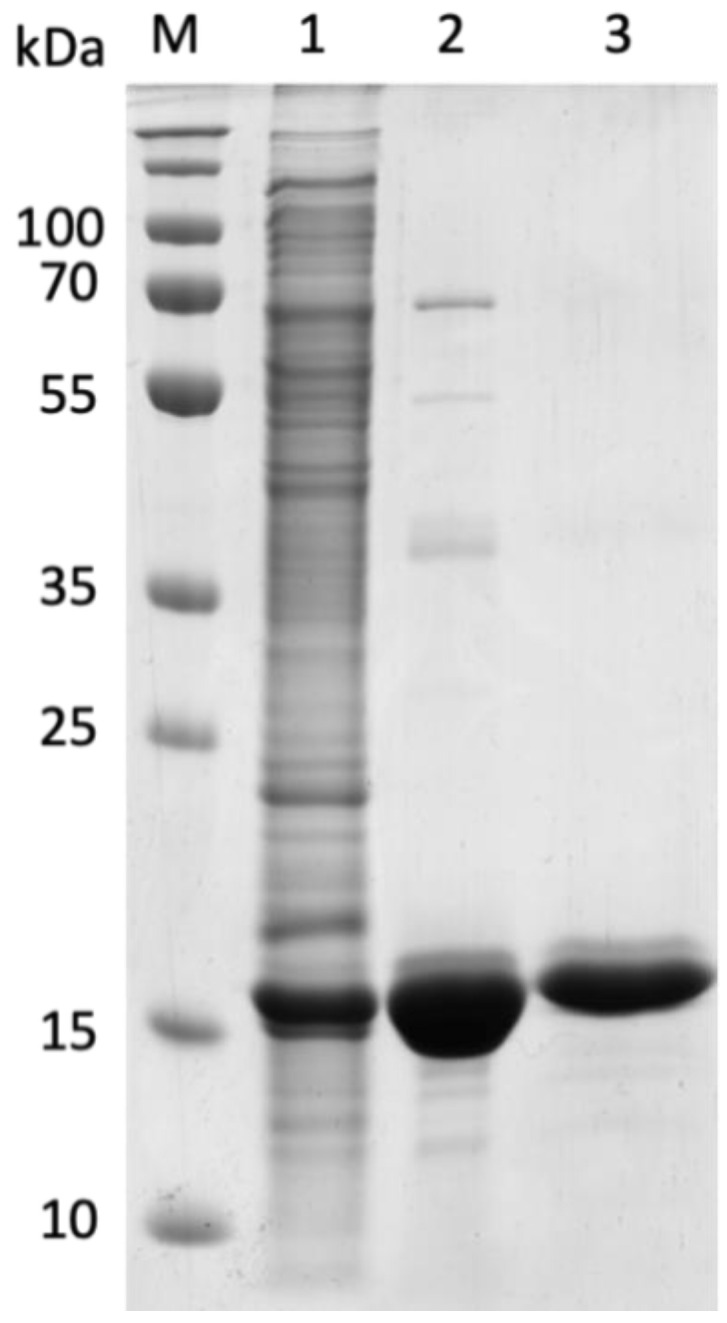
Homologous overexpression and purification of Lrp in *Hfx. mediterranei* HM26-Δ*lrp*. Proteins are shown on a 14.0% SDS-PAGE after Coomassie Brilliant Blue staining. M: PageRuler Plus Prestained Protein Ladder (Thermo Fisher Scientific, Waltham, MA, USA); Line 1: Overexpression of Lrp; Line 2: His-tagged fraction; Line 3: Superose6 Increase 10/300 GL fraction.

As previously explained, a native expression system was used to obtain the Lrp protein [48]. The *lrp* gene was cloned into the pTA1992 plasmid containing an N-terminal hexahistidine (6xHis) tag. The Lrp protein was homologously overexpressed in *Hfx. mediterranei* HM26 and the purification procedure involved two chromatographic steps. The Lrp protein band appeared highly pure in 16 kDa (Figure 4). The purification scheme is summarized in Table 3.

Members of the Lrp/AsnC family are small proteins that typically have a subunit molecular mass between 15 and 17 kDa. In the case of the Lrp protein, the experimental molecular mass in the Superose6 Increase 10/300 GL gel filtration chromatography showed that the most biologically feasible structure of Lrp is a tetrameric protein of approximately 67 kDa. Standard proteins were used as markers (Supplementary Figures S2 and S3) to estimate this result. The theoretical molecular mass of the native Lrp by electrophoresis under denaturing conditions, for the tetramer of 16 kDa per subunit, is 64 kDa. The size of the overexpressed protein was a bit higher than expected due to the SDS-PAGE technique causes an overestimation of the molecular mass of halophilic proteins due to the negative charges [49]. This tetrameric structure is an expected result since Lrp/AsnC transcriptional regulators can form diverse multimers such as dimers, tetramers, octamers and hexadecamers [15,50,51]. An Lrp from the archaeon *Pyrococcus furious* [52] has a tetrameric conformation as the Lrp from this study.

### 3.5. Characterization of lrp Promoter Region Using β-Galactosidase as a Reporter Gene

**Figure 5:**
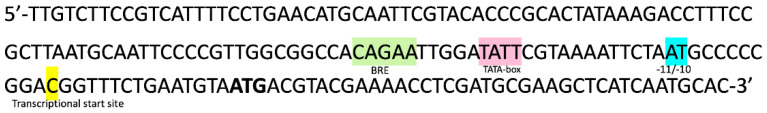
Possible elements of the promoter region. In green the TATA-box; in pink, the BRE-element; in blue, the -11/-10 motif; in yellow, the transcriptional start site; and in bold, the ATG of the *lrp* gene.

**Figure 6:**
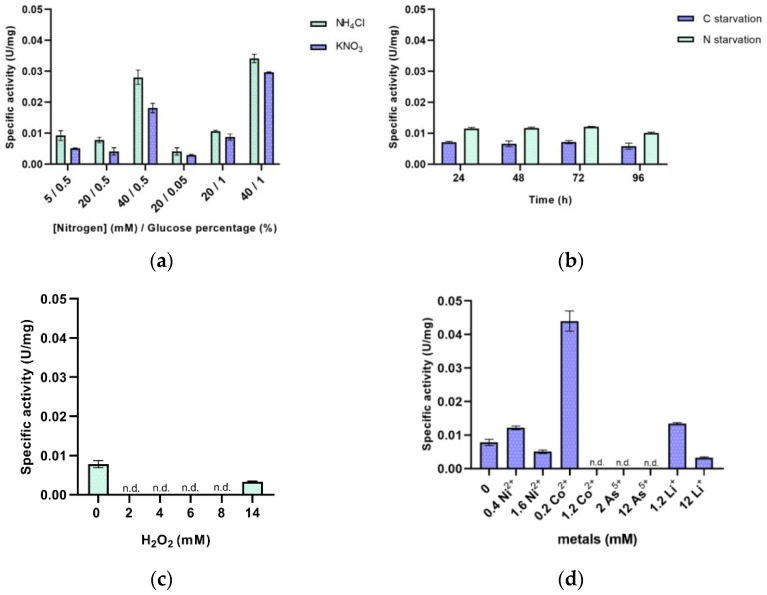
β-galactosidase specific activity of p.*lrp* in cellular extracts from *Hfx. mediterranei* R4 from different culture media. (**a**) Hm-DM containing different concentrations of ammonium or nitrate as the nitrogen source and different concentrations of glucose as the carbon source; (**b**) Hm-NS and Hm-CS at different times of starvation (24, 48, 72 and 96 h); (**c**) Hm-DM with hydrogen peroxide from 0 to 14 mM; (**d**) Hm-DM with Ni^2+^ (0.4 and 1.6 mM), Co^2+^ (0.2 and 1.2 mM), As^5+^ (2 and 12 mM) and Li^+^ (1.2 and 12 mM). n.d. (non detected).

The characterization of the promoter region of the *lrp* gene (Figure 5) [53] was carried out using the *bgaH* gene from *Hfx. lucentense* as a reporter gene by measuring its specific activity using different culture media at the mid-exponential phase. The results are summarized in Figure 5. Remarkably, there are no studies reported comparing the activity of a *lrp* promoter in different media in Haloarchaea. In Hm-CM, the promoter activity was 0.1 U/mg. The specific activity was measured in Hm-DM containing different ammonium or nitrate and glucose concentrations as the nitrogen and carbon sources, respectively (Figure 6A). The best values of specific activity were obtained when cells grew in 40 mM of ammonium or nitrate and 1% glucose. In contrast, the lowest activity was reached with cells grown with 20 mM of ammonium or nitrate and 0.05% of glucose. However, no significant differences in the promoter activity have been identified between cultures shifted to nitrogen or carbon starvation conditions (Figure 6B). Under carbon and nitrogen starvation, there is a low *lrp* expression (around 0.01 U/mg). Therefore, it seems that the *lrp* showed a basal expression at the transcriptional level.

Lrp transcriptional regulators seem to play an essential role in the energy, central metabolism, and coordinating the metabolism in response to environmental alterations. Therefore, culture media adding different external stressors were tested to find a medium where this transcriptional regulator presents changes in its expression to elucidate its function.

On the one hand, there are some evidences about transcriptional factors involved in gene expression regulation under oxidative stress conditions; for example, the MsvR transcriptional factor from *Methanothermobacter thermautotrophicus* regulates the expression of an oxidative stress operon [54]. The ArsR family of transcriptional regulators is usually linked to oxidative conditions because of the existence in their structure of a redox-sensing domain. Moreover, previous investigations in the haloarchaeon *Hbt. salinarum* showed that transcriptional factors Lrp/AsnC were down-regulated in response to oxidative stress conditions after adding 25 mM of hydrogen peroxide (H_2_O_2_) [55]. Therefore, the promoter activity was measured in cell cultures after adding different concentrations of hydrogen peroxide. However, *Hfx. mediterranei* tolerates concentrations lower than *Hbt. salinarum*, cells could not grow above 16 mM H_2_O_2_. For this reason, the tested concentrations were not higher [30]. At low concentrations, between 2 and 8 mM H_2_O_2_, the promoter activity is not detected, while the activity of β-galactosidase slightly increases at higher concentrations (14 mM) of hydrogen peroxide (Figure 6C).

On the other hand, as some transcriptional factors can interact with metal ions, four different heavy metals were added to the culture media to show how the promoter’s activity is affected (Figure 6D). At low concentrations of both, Ni^2+^ and Li^+^, the β-galactosidase specific activity reported was higher than at high concentrations. In contrast to these results, at low concentrations of Co^2+^, the specific activity was detected. However, no activity was reported at higher concentrations. Curiously, in the case of As^5+^, no specific activity was detected at any concentration. The promoter expression is inhibited. With all these results, it could be said that metals may have some effect on the molecular mechanism involved in the expression of *lrp* gene.

In bacteria, the Lrp/AsnC transcriptional regulators only recognize amino acids as ligand molecules, while archaeal Lrp/AsnC proteins may interact with other small molecules as ligands [9]. Although amino acids are the most typical ligands of most characterized Lrp/AsnC transcriptional factors, maybe heavy metals can also act as ligands for this Lrp, being recognized in the ligand-binding domain. This domain is responsible for sensing environmental changes, often interacting with small molecules such as metal ions. The four metals (Li^+,^ Co^2+^, As^5+^, and Ni^2+^) tested in this assay are tolerated by *Hfx. mediterranei* and incorporated into its cellular interior [30]. There is a previous assay about a bacterial heavy-metal resistance system controlled by an Lrp-type transcriptional regulator in *Bacillus subtilis* [56]. For this reason, why not think that these metals may be acting as ligands binding to the Lrp, inducing conformational changes in the structure that may affect the DNA binding by changing the DNA binding affinity? Maybe, the presence of the metal controls the expression of the Lrp.

### 3.6. Western Blot

**Figure 7:**
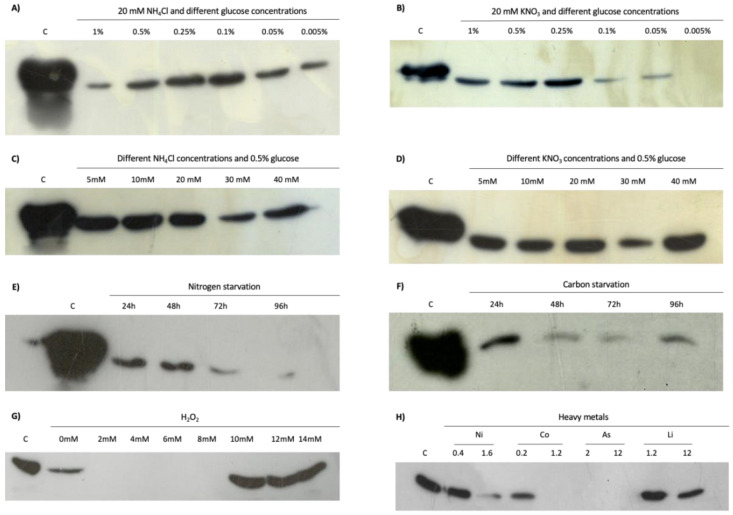
Western blot analysis of the presence of Lrp in cellular extracts from different culture media from *Hfx. mediterranei* R4. (**A**) Hm-DM containing 20 mM of ammonium as the nitrogen source and different concentrations of glucose from 1% to 0.005% as the carbon source.; (**B**) Hm-DM containing 20 mM of nitrate as the nitrogen source and different concentrations of glucose from 1% to 0.005% as the carbon source; (**C**) Hm-DM containing different concentrations of ammonium from 5 to 40 mM as the nitrogen source and 0.5% of glucose as the carbon source; (**D**) Hm-DM containing different concentrations of nitrate from 5 to 40 mM as the nitrogen source and 0.5% of glucose as the carbon source; (**E**) Hm-NS at different times of starvation (24, 48, 72 and 96 h); (**F**) Hm-CS at different times of starvation (24, 48, 72 and 96 h); (**G**) Hm-DM with hydrogen peroxide from 0 to 22 mM; (**H**) Hm-DM with Ni (0.4 and 1.6 mM), Co (0.2 and 1.2 mM), As (2 and 12 mM) and Li (1.2 and 12 mM). 40 µg of the extract was loaded in each condition, and the control has 15 µg of the recombinant homologous protein.

Western blot was performed to analyze in more detail the expression conditions of Lrp in *Hfx. mediterranei* R4, according to the culture media composition. The cell extracts were prepared as previously detailed in Materials and Methods. Results obtained show that Lrp protein is expressed in almost all conditions analyzed (Figure 7). On the one hand, to validate if the glucose concentration influences the expression of this regulator, the samples were collected maintaining the concentration of ammonium or nitrate at 20 mM, but changing the glucose concentration (1%, 0.5%, 0.25%, 0.1%, 0.05%, and 0.005%) (Figure 7A,B). The expression detected adding 20 mM of ammonium as the nitrogen source and different concentrations of glucose as the carbon source was almost identical, indicating that in the presence of ammonium as the nitrogen source, the glucose concentration does not induce any difference in the expression of this transcriptional regulator (Figure 7A). However, maintaining the nitrate concentration at 20 mM and changing the glucose concentration, a decrease in the protein expression was obtained according to the increase of glucose concentration (Figure 7B).

On the other hand, to validate if the amount of nitrogen source influences the expression of the Lrp, culture media with 0.5% glucose as the carbon source and different concentrations of nitrate or ammonium as the nitrogen source were tested (5, 10, 20, 30 and 40 mM). As shown in Figure 7C,D, Western blotting revealed that there is no different amount of Lrp protein in the different samples tested. Furthermore, under carbon or nitrogen deficiency conditions, the detected protein signal is lower (Figure 7E,F). However, these differences may be due to the increase in the number of dead cells. All these results may indicate that the expression of the Lrp is not significantly influenced by the carbon or nitrogen source.

Otherwise, the same stress conditions tested in the β-galactosidase assay were analyzed by Western blot. The amount of the Lrp protein was detected after adding different concentrations of hydrogen peroxide. When the cultures contained low concentrations of H_2_O_2_ (2–8 mM), the Lrp protein was not detected, while above 10 mM the expression appeared (Figure 7G). The addition of heavy metals (Ni^2+^, Li^+^, As^5+^, Co^2+^) was also studied (Figure 7H). At low and high concentrations of both Ni^2+^ and Li^+^, the Lrp was detected. However, at high Co^2+^ concentrations, the protein was not detected, while at low concentrations, the protein was detected. No protein was detected using As^5+^. All these data obtained by Western blot agree with the characterization of its promoter in the previous section. Therefore, it can be considered that this transcriptional regulator has a basal expression due to its implication in different procedures, acting as a global regulator in the transcription. No difference has been found between the level of expression depending on the nitrogen source. Even though this transcriptional regulator recognizes the *nasABC* promoter, an enzyme involved in nitrogen metabolism [57], the Lrp expression may vary depending on the ligands changing their regulation mechanism and having different functions.

## 4. Conclusions

The *Hfx. mediterranei* Lrp transcriptional factor (HFX_RS01210) has been studied in-depth using different approaches to determine its biochemical characteristics and elucidate its expression under different conditions. First, the Lrp has been homologously overexpressed in *Hfx. mediterranei* HM26, employing an expression plasmid developed for halophilic archaea. It is the first overexpression followed by purification of a recombinant protein using *Hfx. mediterranei* as host. The Lrp protein has been purified in its native form by two chromatographic steps, appearing highly pure in SDS-PAGE (16 kDa) and showing that Lrp is a tetrameric protein of approximately 67 kDa, a characteristic structure for most Lrp/AsnC proteins.

It can be deduced that the *lrp* expression is not directly dependent on the nitrogen source, taking into account the data obtained on the characterization of the *lrp* promoter region and the protein expression profile. Although the level of expression does not change, maybe the binding of unknown ligands modulates the activity or the function of the Lrp. Therefore, future assays to elucidate its regulation mechanism will be needed. Interestingly, more relevant results have been obtained by stressing *Hfx. mediterranei*, showing differential expression of Lrp at the transcriptional and translational level. Lrp is expressed in the presence of high concentrations of hydrogen peroxide and the presence of some metals. There was no expression at a high concentration of cobalt and arsenic. Therefore, it can be hypothesized that the binding of ligands is modulating the function of Lrp under these conditions, stabilizing or destabilizing particular types of assemblies. On the basis of these results, it appears that the Lrp could be acting in vivo as a stress regulator of metabolism.

## Figures and Tables

**Table 1:** Culture media used in the β-galactosidase assay.

Description	Culture Media
Complex medium	Hm-CM
	Nitrogen and carbon conditions
Different nitrogen source and concentrations	Hm-DM in the presence of 5–40 mM (5, 20 and 40 mM) ammonium or nitrate as the nitrogen source and 0.5% (*w*/*v*) glucose as the carbon source.
Different carbon source and concentrations	Hm-DM in the presence of 20 mM ammonium or nitrate as the nitrogen source and 0.05–1% (*w*/*v*) (0.05, 0.5 and 1%) glucose as the carbon source.
Starvation of nitrogen	Hm-NS
Starvation of carbon	Hm-CS
	Stress conditions *
Oxidative stress	Hm-DM cultures were grown to OD_600_ of 0.8 (mid-exponential phase) before adding H_2_O_2_ ranging from 2 to 14 mM (2, 4, 6, 8, 10, 12 and 14 mM).
Metal stress	Hm-DM cultures containing 0.4 and 1.6 mM nickel (Ni^2+^); 2 and 12 mM arsenic (As^5+^); 0.2 and 1.2 mM cobalt (Co^2+^); and 1.2 and 12 mM lithium (Li^+^).

* The addition of hydrogen peroxide and heavy metals were performed as [30]. All the media were inoculated at OD_600_ 0.02 with pre-adapted cells. Three independent biological replicates of each condition were performed, and all the cultures contained 0.3 µg/mL novobiocin.

**Table 2:** Proteins annotated as Lrp/AsnC in halophilic archaeal families and their protein domain structures.

		Domain				
Families *	Number of Lrp	HTH + Ligand-Binding	HTH	Ligand - Binding	HTH + TRASH	HTH + TrkA_C
*Haloarculaceae*	426	63.7%	15%	19.7%	1.4%	0.2%
*Halobacteriaceae*	344	62.8%	10.8%	19.8%	3.5%	3.1%
*Halococcaceae*	48	91.9%	5.4%	2.7%	-	-
*Haloferacaceae*	818	62.9%	15.7%	17.2%	1.9%	2.3%
*Halorubraceae*	594	88.3%	4.5%	4.8%	0.7%	0.7%
*Natrialbaceae*	731	60.2%	16.2%	21.4%	1.4%	0.2%

* Genus studied in each family and number of Lrp/AsnC proteins are shown in Table S1.

**Table 3:** Description of the purification steps for Lrp from *Hfx. mediterranei*.

	Volume (mL)	Protein (mg/mL)	Yield (%)
**Overexpressed extract**	25	13.89	100
**His-tagged fraction**	5	9.787	14.1
**Superose6 Increase 10/300 GL**	5	1.659	2.4

## Data Availability

The data presented in this study are available within the article.

## Supplementary Materials

The following are available online at https://www.mdpi.com/article/10.3390/genes12060802/s1, Figure S1: EMBOSS Needle alignment of Lrp (*Hfx. mediterranei*) with Lrp (*Hbt. salinarum*), Figure S2: Chromatogram of standard proteins (dotted line) and Lrp (red line) using Superose6 Increase 10/300GL column., Figure S3: Size-exclusion chromatography calibration curve for Superose6 increase 10/300GL column, Table S1: Genus studied in each family and number of annotated Lrp/AsnC proteins.

## References

[1] Oren A. (2002). Molecular ecology of extremely halophilic Archaea and Bacteria. FEMS Microbiol. Ecol..

[2] Andrei A.-S., Banciu H.L., Oren A. (2012). Living with salt: Metabolic and phylogenetic diversity of archaea inhabiting saline ecosystems. FEMS Microbiol. Lett..

[3] Cabrera M. (2018). Ángeles; Blamey, J.M. Biotechnological applications of archaeal enzymes from extreme environments. Biol. Res..

[4] Bonete M.-J., Martínez-Espinosa R.M., Pire C., Zafrilla B., Richardson D.J. (2008). Nitrogen metabolism in haloarchaea. Saline Syst..

[5] Koike H., Ishijima S.A., Clowney L., Suzuki M. (2004). The archaeal feast/famine regulatory protein: Potential roles of its assembly forms for regulating transcription. Proc. Natl. Acad. Sci. USA.

[6] Lemmens L., Maklad H.R., Bervoets I., Peeters E. (2019). Transcription Regulators in Archaea: Homologies and Differences with Bacterial Regulators. J. Mol. Biol..

[7] Pérez-Rueda E., Janga S.C. (2010). Identification and Genomic Analysis of Transcription Factors in Archaeal Genomes Exemplifies Their Functional Architecture and Evolutionary Origin. Mol. Biol. Evol..

[8] Brinkman A.B., Ettema T.J.G., de Vos W.M., van der Oost J. (2003). The Lrp family of transcriptional regulators. Mol. Microbiol..

[9] Peeters E., Charlier D. (2010). The Lrp Family of Transcription Regulators in Archaea. Archaea.

[10] Tani T.H., Khodursky A., Blumenthal R.M., Brown P.O., Matthews R.G. (2002). Adaptation to famine: A family of stationary-phase genes revealed by microarray analysis. Proc. Natl. Acad. Sci. USA.

[11] Charlier D., Roovers M., Thia-Toong T.-L., Durbecq V., Glansdorff N. (1997). Cloning and identification of the Sulfolobus solfataricus lrp gene encoding an archaeal homologue of the eubacterial leucine-responsive global transcriptional regulator Lrp. Gene.

[12] Schwaiger R., Schwarz C., Furtwängler K., Tarasov V., Wende A., Oesterhelt D. (2010). Transcriptional control by two leucine-responsive regulatory proteins in Halobacterium salinarum R. BMC Mol. Biol..

[13] Vassart A., van Wolferen M., Orell A., Hong Y., Peeters E., Albers S., Charlier D. (2012). Sa- L rp from *Sulfolobus acidocaldarius* is a versatile, glutamine-responsive, and architectural transcriptional regulator. MicrobiologyOpen.

[14] Ouhammouch M., Geiduschek E. (2001). A thermostable platform for transcriptional regulation: The DNA-binding properties of two Lrp homologs from the hyperthermophilic archaeon *Methanococcus jannaschii*. EMBO J..

[15] Leonard P.M., Smits S.H., Sedelnikova S.E., Brinkman A.B., de Vos W.M., van der Oost J., Rice D.W., Rafferty J.B. (2001). Crystal structure of the Lrp-like transcriptional regulator from the archaeon *Pyrococcus furiosus*. EMBO J..

[16] Martãnez-Espinosa R.M., Esclapez J., Bautista V., Bonete M.J., Martínez-Espinosa R.M., Bonete M.J. (2006). An octameric prokaryotic glutamine synthetase from the haloarchaeon *Haloferax mediterranei*. FEMS Microbiol. Lett..

[17] Rodríguez-Herrero V., Payá G., Bautista V., Vegara A., Cortés-Molina M., Camacho M., Esclapez J., Bonete M.J. (2020). Essentiality of the glnA gene in *Haloferax mediterranei*: Gene conversion and transcriptional analysis. Extremophiles.

[18] Sievers F., Wilm A., Dineen D., Gibson T.J., Karplus K., Li W., Lopez R., McWilliam H., Remmert M., Söding J. (2011). Fast, scalable generation of high-quality protein multiple sequence alignments using Clustal Omega. Mol. Syst. Biol..

[19] Söding J. (2005). Protein homology detection by HMM-HMM comparison. Bioinformation.

[20] Letunic I., Bork P. (2019). Interactive Tree of Life (iTOL) v4: Recent updates and new developments. Nucleic Acids Res..

[21] Pedro-Roig L., Lange C., Bonete M.J., Soppa J., Maupin-Furlow J. (2013). Nitrogen regulation of protein-protein interactions and transcript levels of GlnK PII regulator and AmtB ammonium transporter homologs in Archaea. MicrobiologyOpen.

[22] Rodriguez-Valera F., Juez G., Kushner D. (1983). *Halobacterium mediterranei* spec, nov., a New Carbohydrate-Utilizing Extreme Halophile. Syst. Appl. Microbiol..

[23] Haque R.U., Paradisi F., Allers T. (2020). *Haloferax volcanii* for biotechnology applications: Challenges, current state and perspectives. Appl. Microbiol. Biotechnol..

[24] Haque R.U., Paradisi F., Allers T. (2019). *Haloferax volcanii* as immobilised whole cell biocatalyst: New applications for halophilic systems. Appl. Microbiol. Biotechnol..

[25] Holmes M.L., Scopes R.K., Moritz R.L., Simpson R.J., Englert C., Pfeifer F., Dyall-Smith M.L. (1997). Purification and analysis of an extremely halophilic β-galactosidase from *Haloferax alicantei*. Biochim. Biophys. Acta Protein Struct. Mol. Enzym..

[26] Serrano-Gomicia J.A. (2000). Ciclo del glioxilato en el arquea halófilo *Haloferax volcanii*: Análisis bioquímico, filogenético y transcripcional. Ph.D. Thesis.

[27] Dower W.J., Miller J.F., Ragsdale C.W. (1988). High efficiency transformation of *E. coli* by high voltage electroporation. Nucleic Acids Res..

[28] Cline S.W., Lam W.L., Charlebois R.L., Schalkwyk L.C., Doolittle W.F. (1989). Transformation methods for halophilic archaebacteria. Can. J. Microbiol..

[29] Bradford M.M. (1976). A rapid and sensitive method for the quantitation of microgram quantities of protein utilizing the principle of protein-dye binding. Anal. Biochem..

[30] Matarredona L., Camacho M., Zafrilla B., Bravo-Barrales G., Esclapez J., Bonete M.-J. (2021). The Survival of *Haloferax mediterranei* under Stressful Conditions. Microorganisms.

[31] Oren A., Hallsworth J.E. (2014). Microbial weeds in hypersaline habitats: The enigma of the weed-like *Haloferax mediterranei*. FEMS Microbiol. Lett..

[32] Harris T.J., Emtage J.S. (1986). Expression of heterologous genes in E. coli. Microbiol. Sci..

[33] Jia B., Jeon C.O. (2016). High-throughput recombinant protein expression in *Escherichia coli*: Current status and future perspectives. Open Biol..

[34] Gileadi O. (2017). Recombinant Protein Expression in *E. coli*: A Historical Perspective. Methods in Molecular Biology.

[35] Mevarech M., Frolow F., Gloss L.M. (2000). Halophilic enzymes: Proteins with a grain of salt. Biophys. Chem..

[36] Danson M.J., Hough D.W. (1997). The Structural Basis of Protein Halophilicity. Comp. Biochem. Physiol. Part A Physiol..

[37] Britton K.L., Baker P.J., Fisher M., Ruzheinikov S., Gilmour D.J., Bonete M.-J., Ferrer J., Pire C., Esclapez J., Rice D.W. (2006). Analysis of protein solvent interactions in glucose dehydrogenase from the extreme halophile *Haloferax mediterranei*. Proc. Natl. Acad. Sci. USA.

[38] Domenech J., Ferrer J. (2006). A new d-2-hydroxyacid dehydrogenase with dual coenzyme-specificity from Haloferax mediterranei, sequence analysis and heterologous overexpression. Biochim. Biophys. Acta Gen. Subj..

[39] Pire C., Esclapez J., Ferrer J., Bonete M.-J. (2001). Heterologous overexpression of glucose dehydrogenase from the halophilic archaeonHaloferax mediterranei, an enzyme of the medium chain dehydrogenase/reductase family. FEMS Microbiol. Lett..

[40] Diaz S., Pérez-Pomares F., Pire C., Ferrer J., Bonete M.-J. (2005). Gene cloning, heterologous overexpression and optimized refolding of the NAD-glutamate dehydrogenase from *Haloferax mediterranei*. Extremophiles.

[41] Pérez-Pomares F., Bautista V., Ferrer J., Pire C., Marhuenda-Egea F.C., Bonete M.-J., Marhuenda-Egea F. (2003). α-Amylase activity from the halophilic archaeon *Haloferax mediterranei*. Extremophiles.

[42] Camacho M., Rodríguez-Arnedo A., Bonete M.-J. (2002). NADP-dependent isocitrate dehydrogenase from the halophilic archaeonHaloferax volcanii: Cloning, sequence determination and overexpression in *Escherichia coli*. FEMS Microbiol. Lett..

[43] Esclapez J., Bonete M.J., Camacho M., Pire C., Ferrer J., Bautista V., Martínez-Espinosa R.M., Zafrilla B., Pérez-Pomares F., Díaz S. (2006). An optimized method to produce halophilic proteins in Escherichia coli. Microb. Cell Fact..

[44] Jolley K.A., Rapaport E., Hough D.W., Danson M.J., Woods W.G., Dyall-Smith M.L. (1996). Dihydrolipoamide dehydrogenase from the halophilic archaeon *Haloferax volcanii*: Homologous overexpression of the cloned gene. J. Bacteriol..

[45] Fine A., Irihimovitch V., Dahan I., Konrad Z., Eichler J. (2006). Cloning, Expression, and Purification of Functional Sec11a and Sec11b, Type I Signal Peptidases of the Archaeon *Haloferax volcanii*. J. Bacteriol..

[46] Timpson L.M., Liliensiek A.-K., Alsafadi D., Cassidy J., Sharkey M.A., Liddell S., Allers T., Paradisi F. (2013). A comparison of two novel alcohol dehydrogenase enzymes (ADH1 and ADH2) from the extreme halophile *Haloferax volcanii*. Appl. Microbiol. Biotechnol..

[47] Esclapez J., Zafrilla B., Martínez-Espinosa R.M., Bonete M.-J. (2013). Cu-NirK from Haloferax mediterranei as an example of metalloprotein maturation and exportation via Tat system. Biochim. Biophys. Acta Proteins Proteom..

[48] Allers T., Barak S., Liddell S., Wardell K., Mevarech M. (2010). Improved Strains and Plasmid Vectors for Conditional Overexpression of His-Tagged Proteins in *Haloferax volcanii*. Appl. Environ. Microbiol..

[49] Bonete M.-J., Pire C., Llorca F.I., Camacho M.L. (1996). Glucose dehydrogenase from the halophilic Archaeon *Haloferax mediterranei*: Enzyme purification, characterisation and N-terminal sequence. FEBS Lett..

[50] Reddy M.C., Gokulan K., Jacobs W., Ioerger T.R., Sacchettini J.C. (2007). Crystal structure of *Mycobacterium tuberculosis* LrpA, a leucine-responsive global regulator associated with starvation response. Protein Sci..

[51] Thaw P. (2006). Structural insight into gene transcriptional regulation and effector binding by the Lrp/AsnC family. Nucleic Acids Res..

[52] Brinkman A.B., Dahlke I., Tuininga J.E., Lammers T., Dumay V., de Heus E., Lebbink J.H., Thomm M., de Vos W.M., van der Oost J. (2000). An Lrp-like Transcriptional Regulator from the Archaeon *Pyrococcus furiosus* Is Negatively Autoregulated. J. Biol. Chem..

[53] Brenneis M., Hering O., Lange C., Soppa J. (2007). Experimental Characterization of Cis-Acting Elements Important for Translation and Transcription in *Halophilic archaea*. PLoS Genet..

[54] Karr E.A. (2010). The Methanogen-Specific Transcription Factor MsvR Regulates the fpaA-rlp-rub Oxidative Stress Operon Adjacent to msvR in *Methanothermobacter thermautotrophicus*. J. Bacteriol..

[55] Plaisier C.L., Lo F.-Y., Ashworth J., Brooks A.N., Beer K.D., Kaur A., Pan M., Reiss D.J., Facciotti M.T., Baliga N.S. (2014). Evolution of context dependent regulation by expansion of feast/famine regulatory proteins. BMC Syst. Biol..

[56] Aguilar-Barajas E., Jacobo-Arreola S., Verduzco-Rosas L.A., Jiménez-Mejía R., Ramírez-Díaz M.I., Julián-Sánchez A., Riveros-Rosas H., Cervantes C. (2013). An Lrp-type transcriptional regulator controls expression of the Bacillus subtilis chromate transporter. Antonie Leeuwenhoek Int. J. Gen. Mol. Microbiol..

[57] Pastor-Soler S., Camacho M., Bautista V., Bonete M.-J., Esclapez J. (2021). Towards the Elucidation of Assimilative *nasABC* Operon Transcriptional Regulation in *Haloferax mediterranei*. Genes.

