# Supplementary material for: Analysis of Haloferax mediterranei Lrp Transcriptional Regulator
Source: Genes (Basel). 2021 May 25;12(6):802. doi: 10.3390/genes12060802 (PMC8229911; doi:10.3390/genes12060802)
Supplement: Supplementary file 1 [file genes-12-00802-s001.zip › genes-1219619-supplementary.pdf]

Table S1. Genus studied in each family and number of annotated Lrp/AsnC proteins. In parenthesis the number of Lrp/AsnC proteins.

Families	***Genus***
Haloarculaceae (426)	*Halapricum* (15); *Haloarcula* (233); *Halomicrobium* (36); *Halorhabdus* (25); *Halorientalis* (40); *Halosimplex* (8); *Natronomonas* (69)
Halobacteriaceae (344)	*Haladaptatus* (58); *Halalkalicoccus* (13); *Halanaeroarchaeum* (5); *Halarchaeum* (17); *Halobacterium* (174); *Halodesulfurarchaeum* (7); *Halomarina* (11); *Halovenus* (17); *Natronoarchaeum* (11); *Salarchaeum* (8); *Halorussus* (9); *Haloarchaeobius* (14)
Halococcaceae (48)	*Halococcus* (48)
Haloferacaceae (818)	*Halobellus* (49); *Haloferax* (546); *Halogeometricum* (64); *Halogranum* (46); *Halopelagius* (27); *Haloplanus* (40); *Haloprofundus* (17); *Haloquadratum* (29)
Halorubraceae (594)	*Halobium* (12); *Halohasta* (15); *Halolamina* (21); *Halonotius* (36); *Halopenitus* (28); *Halorubrum* (444); *Salinigranum* (21); *Halobaculum* (17)
Natrialbaceae (721)	*Halobiforma* (27); *Halopiger* (37); *Halostagnicola* (29); *Haloterrigena* (98); Halovivax (11); *Natrarchaeobius* (33); *Natrialba* (91); *Natrinema* (127); *Natronobacterium* (51); *Natronococcus* (35); *Natronolimnobius* (64); *Natronorubrum* (121); *Salinarchaeum* (7)

```
Hfx. mediterranei     1 MTYENLDAKLINALLGDGRASLRSLAEELDVSVTTVSNHLRDLEDEGVIE     50
                        |||||||.||:|.|||||||||||||::||||||||||||:.|||||.:.
Hbt. salinarum        1 MTYENLDVKLVNELLGDGRASLRSLADDLDVSVTTVSNHLQTLEDEGAVN     50

Hfx. mediterranei    51 GYTPRVNYDALGYDVTAVIQLKVEGSALPEITERLRAEKQMISVYEVTGD    100
                        ||||.|:|:.|||||||::||||:|:||||||..||..|||:||||:|||
Hbt. salinarum       51 GYTPVVDYERLGYDVTAILQLKVDGTALPEITGTLRGHKQMVSVYEITGD    100

Hfx. mediterranei   101 YDIIAIGKFRDTDGMNTQIKKLLTDTDIRESNTSVVLNAVTENEQFALDV    150
                        ||::|||||.|||.|||.||:||.|.||.||:|||||||..|||||.||:
Hbt. salinarum      101 YDVLAIGKFTDTDDMNTLIKELLADADINESSTSVVLNAAAENEQFELDL    150

Hfx. mediterranei   151 DE--      152
                        |.
Hbt. salinarum      151 DGDA      154
```

Figure S1. EMBOSS Needle alignment of Lrp (*Hfx. mediterranei*) with Lrp (*Hbt. salinarum*). The HTH binding domain is shaded in pink.

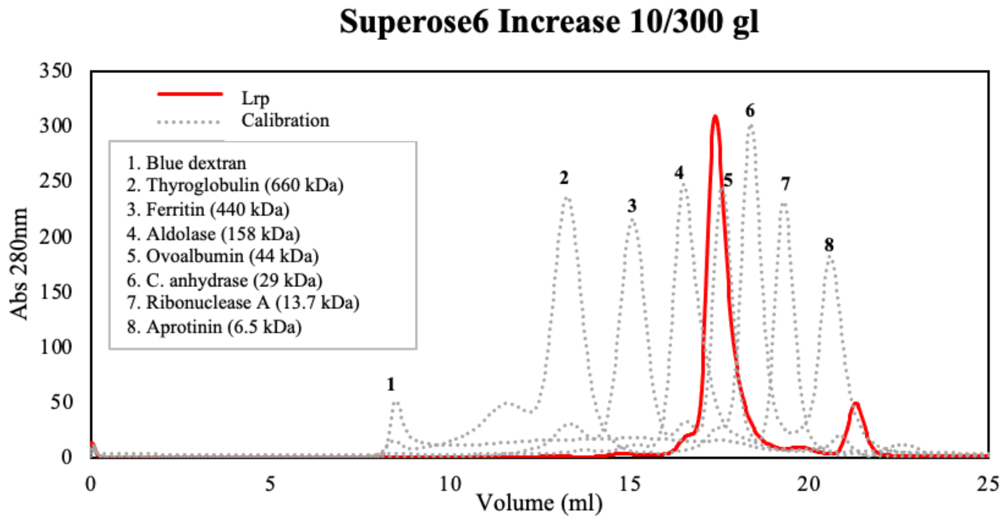

Figure S2. Chromatogram of standard proteins (dotted line) and Lrp (red line) using Superose6 Increase 10/300GL column.

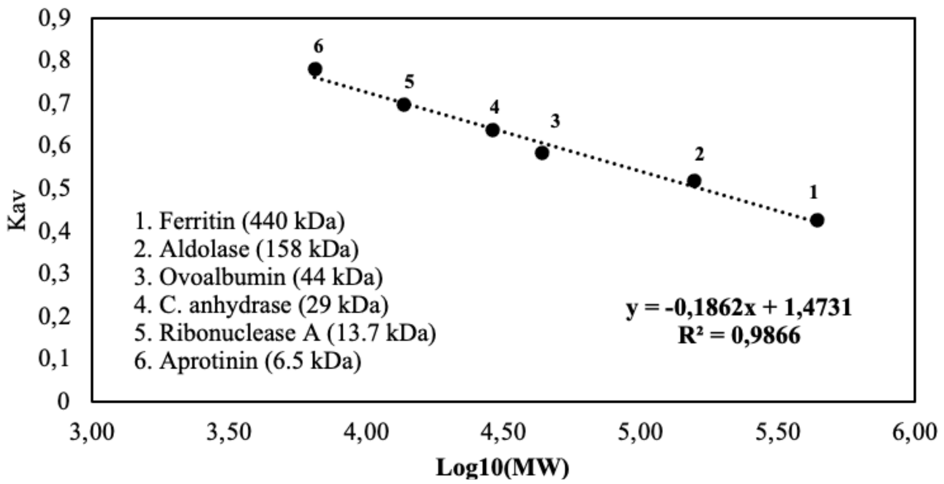

Figure S3. Size exclusion chromatography calibration curve for Superose6 Increase 10/300GL column.

.
